# Community State Types of Vaginal Microbiota and Four Types of Abnormal Vaginal Microbiota in Pregnant Korean Women

**DOI:** 10.3389/fpubh.2020.507024

**Published:** 2020-10-22

**Authors:** Sunghee Lee, Kwan Young Oh, Heeji Hong, Chan Hee Jin, Eunjung Shim, Seung Hyun Kim, Byung-Yong Kim

**Affiliations:** ^1^Research Laboratories, ILDONG Pharmaceutical Co., Ltd., Hwaseong-si, South Korea; ^2^Department of Obstetrics and Gynecology, School of Medicine, Eulji University, Daejeon, South Korea; ^3^Microbiome Research Center, ChunLab, Inc., Seocho-gu, South Korea

**Keywords:** bacterial vaginosis, intermediate flora, abnormal vaginal flora, next-generation sequencing, pregnant Korean women, *Lactobacillus* spp., *Gardnerella vaginalis*, *Bifidobacterium breve*

## Abstract

Abnormal vaginal microbiota (AVM), including bacterial vaginosis (BV), is caused by a microbiota imbalance. Nugent scoring is the gold standard for the laboratory diagnosis of BV; however, it is somewhat subjective to interpret, and challenging to distinguish bacteria. Hence, there is a need for improved technologies for the accurate diagnosis of AVM. To this end, next-generation sequencing (NGS) technology has been shown to yield comprehensive information on the pathophysiology of AVM. Hence, to evaluate the relationship between microbiota composition and the pathophysiology of AVM and its clinical significance, we characterized vaginal swab samples from 212 pregnant Korean women using both Nugent scoring and NGS analysis. Of these, the Nugent scoring identified 175 subjects (82.5%; 175/212) with normal flora (NF), 20 (9.4%; 20/212) with intermediate flora (IF), and 17 (8.0%; 17/212) with BV. NGS analysis followed by the characterization of vaginal microbiota composition, as represented by alpha and beta diversity, revealed the relative abundance of specific bacterial taxa at the genus and species level. Moreover, we identified all five predominant community state types (CSTs) along with three smaller CSTs. Analysis of the vaginal microbiota revealed the dominance of one or two *Lactobacillus* spp. in the NF group. Meanwhile, the IF and BV groups were dominated by the genera *Gardnerella, Prevotella*, and *Atopobium*. These two groups also showed higher alpha diversity than the NF group (*p* < 0.05). Principal coordinate analysis (PCoA) indicated that the NF group was significantly different from the AVM groups (*p* < 0.05), whereas no significant difference was observed between IF and BV groups (*p* = 0.25). Lastly, to investigate the characteristics of vaginal microbiota based on taxonomic composition, the IF and BV groups (AVM groups) were reclassified using the unweighted pair group method with arithmetic mean (UPGMA) clustering. Consequently, they were reclassified into BV1 *(Lactobacillus iners*-dominated), BV2-1 (*Bifidobacterium breve*-dominated), BV2-2 (*Gardnerella vaginalis* s1 or s2 and *Atopobium vaginae*-dominated), and BV3 [mixed population of *G. vaginalis, L. iners*, and other bacteria (*p* < 0.05)]. Collectively, these findings could serve to advance the current understanding regarding AVM pathophysiology.

## Introduction

Vaginal microbiota are associated with women's reproductive health, particularly during pregnancy (1–4). Most of the vaginal microbiota tend to become imbalanced when *Lactobacillus* levels are reduced, which can lead to bacterial vaginosis (BV) (5). BV in pregnant women significantly contributes to poor perinatal outcomes, including miscarriage and preterm birth (6). However, the diagnostic tools for BV are imprecise owing to an inadequate understanding of its pathophysiology. Currently, the Nugent scoring and Amsel's criteria are used to diagnose BV (7–9). Amsel's criteria are composed of four criteria: increased thin homogenous vaginal discharge, amine odor under whiff test, vaginal pH > 4.5, and the presence of clue cells (8). Srinivasan et al. (10) reported that each of these criteria was associated with specific microorganisms such as *Gardnerella vaginalis, Atopobium vaginae*, and *Leptotrichia amnionii*. The Nugent score is the gold standard for BV diagnosis (9); however, it has certain limitations: (i) intermediate flora (IF) cannot be readily interpreted for clinical significance, and (ii) symptomatic and asymptomatic women with BV cannot be differentiated (11, 12). Alternatively, the next-generation sequencing (NGS) diagnostic methods have gained popularity. It has been shown that deep sequencing and species level taxonomic classification can distinguish bacterial species, and hence, is useful to understand microbiota compositions and the pathogenesis of BV (10, 13). Furthermore, the vaginal microbiota composition can vary with different factors such as pregnancy, ethnicity, and residential areas (14–19). Though several high-throughput vaginal microbiota studies have been reported, they have primarily focused on Caucasian cohorts in the United States and Europe, whereas very few have focused on pregnant Korean women to determine their vaginal microbiome composition such as community state types (CSTs).

Therefore, to precisely evaluate the relationship between the vaginal microbial composition of pregnant Korean women and the pathophysiology of abnormal vaginal microbiota (AVM), as well as its clinical significance, herein we aimed to characterize vaginal swab samples from 212 pregnant Korean women using both Nugent scoring and NGS analysis.

## Materials and Methods

### Study Design

This was a cross-sectional study that sought to analyze the relationship between vaginal microbial composition and the pathophysiology of AVM, as well as its clinical significance, including in preterm birth. The sample size was calculated based on the rate of preterm birth (~10% at Eulji University Hospital). Therefore, since the number of expected preterm birth cases in women who received antenatal care from the first trimester in Eulji University Hospital within a year was 25, we sought to enroll 250 pregnant women in the late first trimester (10–14 weeks of gestation). The gestational age was decided based on several studies reporting that preterm birth is preventable via treatment of BV in the first trimester (20–22). Furthermore, 10–14 weeks of gestation was chosen to exclude other causes of abortion besides vaginal infection.

### Selection Criteria

Pregnant women under antenatal care at Eulji University Hospital and local clinics (i.e., Seoul Women's Hospital) were enrolled at 10–14 weeks gestation. Women who received antibiotics or vaginal suppositories within the previous 2 weeks, as well as those with a preexisting medical condition (i.e., chronic hypertensive disease, hyperthyroidism, or other diseases), were excluded. Ultimately, a total of 228 pregnant Korean women were enrolled, 174 of whom were enrolled from the Department of Obstetrics and Gynecology at Eulji University Hospital, and 54 were enrolled from local clinics.

After obtaining written consent, the symptoms, including an increase in thin homogenous vaginal discharge and foul fish-like odors (we did not conduct the whiff test) were recorded for each subject via a questionnaire. In addition, the history of preterm birth, abortion, and general obstetric history, as well as the presence or absence of existing genital infections were recorded. Women diagnosed with BV based on their Nugent score and clinical symptoms were treated with clindamycin. Perinatal outcomes such as the delivery week, birth weight, and obstetric complications were reviewed from medical records. After initial enrolment, a total of 16 women were excluded from analysis due to reported obstetric or medical illness (preeclampsia, five cases; gestational diabetes, seven cases; other medical diseases, including gastrointestinal disease, four cases), resulting in 212 women participating in the study. This study was approved by the Institutional Review Board (IRB) of Eulji University Hospital (IRB no.: 2017-07-007-002).

### Vaginal Sampling

Samples from the enrolled subjects were collected by a physician using a cotton swab for Gram staining and ESwab (Copan, Italia) for NGS analysis, from the posterior fornix of the vaginal wall. The vaginal pH was measured by inserting the Litmus tape into the posterior fornix of the vaginal wall, and the observations were interpreted according to the color guide by a clinician after sampling. The cotton swab was then smeared on a slide, dried to fix the sample, and sent to the laboratory for Gram staining. The samples for NGS analysis were transferred to the laboratory within 10 min after collection and stored in a freezer at −80°C until use.

### Diagnosis of Abnormal Vaginal Microbiota

The Nugent scoring based on Gram staining was used to diagnose the BV and IF (9). Nugent scores were obtained for *Lactobacillus* like, *Mobiluncus* like, *Gardnerella* like, and *Bacteroides* like morphotypes on Gram-stained slides (9), and based on the scores, the subjects were classified into three groups: BV, IF, and NF. We also evaluated the presence of clue cells through microscopic observation of the slides. These scores were performed twice by a professor and a technician in the laboratory department and cross-checked for validation.

### Next-Generation Sequencing Analysis

Samples stored at −80°C were defrosted at room temperature (20–25°C) for 30 min and vortexed in a FastPrep®-24 instrument (MP Biomedicals, USA) by adding 200 μL of samples to the PowerBead Tubes (Garnet 0.79 mm, Qiagen, Helden, Germany) to homogenize each sample. DNA was then extracted from these samples using the QIAamp DNA Mini QIAcube Kit (#51326, Qiagen, Hilden, Germany). The amount of isolated DNA was measured using a NanoDrop apparatus (NanoDrop Technologies, Inc., Wilmington, DE, USA) and stored at −80°C until NGS analysis. The genomic DNA was amplified by polymerase chain reaction (PCR) using a universal primer set (341F: 5′-TCGTCGGCAGCGTCAGATGTGTATAAGAGACAG*CCTACGGGNGGCWGCAG*-3′, and 805R: 5′-GTCTCGTGGGCTCGGAGATGTGTATAAGAGACAG*GACTACHVGG GTATCTAAT CC*-3′, (the underlined sequence indicates the target region) (23) with Illumina overhang adaptors targeting the V3–V4 region of the 16S rRNA gene. PCR amplification was performed using the following thermal cycling conditions: initial denaturation at 95°C for 3 min, followed by 25 cycles of denaturation at 95°C for 30 s, primer annealing at 55°C for 30 s, and extension at 72°C for 30 s, with a final elongation step at 72°C for 5 min. Secondary amplification for attaching the Illumina Nextera barcode was performed with the i5 forward and i7 reverse primer (i5: 5′-AATGATACGGCGACCACCGAGATCTACAC-XXXXXXXX-TCGTCGGCAGCGTC-3′; i7: 5′-CAAGCAGAAGACGGCATACGAGAT-XXXXXXXX-GTCTCGTGGGCTCGG-3′; X indicates the barcode region). The amplification conditions were similar to those described above, except that the amplification cycle was set to eight cycles. The PCR product was confirmed using 1.0% agarose gel electrophoresis and visualized under a Gel Doc system (BioRad, Hercules, CA, USA). The amplified products were purified using the CleanPCR kit (CleanNA, Inc., Netherlands). Equal concentrations of purified products were pooled together, and short fragments (non-target products) were removed using the CleanPCR kit. The DNA quality and product size were assessed on a Bioanalyzer 2100 (Agilent, Palo Alto, CA, USA) using a DNA 7500 chip. Mixed amplicons were pooled, and sequencing was carried out at ChunLab, Inc. (Seoul, Korea) using the Illumina MiSeq Sequencing System (Illumina, USA), according to the manufacturer's instructions.

### Bioinformatics Analyses

De-multiplexed paired-end reads obtained from the MiSeq platform were imported into the Quantitative Insights into Microbial Ecology 2 (QIIME 2, ver.2018.6.0) analysis pipeline using the FASTQ manifest protocol (24). Primers in the raw sequences were trimmed using Cutadapt (25). The paired-end reads were then merged using the Vsearch merge pairs function (26). The resulting merged reads were then filtered to exclude the low-quality reads based on a median quality score of Q25, and ambiguous base calls, as well as all chimeric sequences, were removed using the Deblur workflow (27).

Multiple alignments of the resulting sequences were performed using MAFFT ver. 7, a method suitable for multiple alignments of a large number of short sequences (28). Uninformative base positions based on the lane mask were then removed, and the resulting aligned sequences were used to generate a phylogenetic tree using the FastTree algorithm (29, 30). The EzBioCloud database, which is complementary to QIIME 2, was used for taxonomic analysis based on 80% identity using the BLAST+ consensus taxonomy classifier (31, 32). The relative abundance (RA) of the top 30 operative taxonomic units (OTUs) in the pregnant Korean women were calculated and represented using a heatmap, created using Multiple Experiment Viewer (MeV) software (ver. 4.9.0, http://www.tigr.org/software/tm4~/mev.html).

Alpha and beta diversity metrics were extracted using the core-metrics-phylogenetic function, based on phylogenetic and non-phylogenetic trees. Species richness was compared based on the Faith-Phylogenetic diversity (Faith's PD), Shannon index, and the number of OTUs (33, 34). The overall phylogenetic distance among different groups was estimated using the weighted UniFrac dissimilarity based on the phylogenetic tree (35, 36). After NGS analysis, we reclassified the subjects diagnosed with BV and IF (constituting the AVM group) using the unweighted pair group method with arithmetic mean (UPGMA) clustering to investigate the characteristics of vaginal microbiota based on its species level composition.

### Statistical Analysis

Demographic factors were compared among the three groups (BV, IF, and NF groups) using the non-parametric Kruskal–Wallis test followed by the Mann–Whitney *U*-test. RA and alpha diversity were analyzed to compare the quantitative differences among the classified and reclassified AVM groups. Statistical significance of the differences for RA and alpha diversity was tested using the non-parametric Kruskal–Wallis test, followed by the Mann–Whitney *U*-test with *p* < 0.05. Bonferroni's correction test was applied as a correction for multiple comparisons. All statistical analyses were carried out using SPSS version 18 (SPSS, Chicago, IL, USA). Besides, RA was further analyzed using the linear discriminant analysis effect size (LEfSe) program to explore the potential presence of taxonomic clades that can serve as biomarkers for different classes. Statistically significant groups were reported with linear discriminant analysis (LDA) scores >4 (37). For beta diversity, the significant differences among groups were assessed by permutation-based multivariable analysis of variance (PERMANOVA) testing, which was performed with a plug-in of QIIME2, with 10,000 replicates.

## Results

### Demographic Characteristics of the Samples

The Nugent scoring of the samples from these 212 women identified 175 (82.5%; 175/212) women with NF, 20 (9.4%; 20/212) with IF, and 17 (8.0%; 17/212) with BV. The differences for various demographic factors such as maternal age, parity, abortion history, and rate of previous preterm birth among the three groups were found to be statistically insignificant (Table 1). Vaginal pH was observed to be significantly increased in the IF and BV groups compared to that in the NF group *(p* = 0.047). However, the perinatal outcomes, such as delivery weeks, and rate of preterm birth, were not significantly different (*p* > 0.05; Table 1).

**Table 1:** Demographic characteristics of the three groups using the Nugent scoring system.

**Demographic characteristics**	**NF group (*n* = 175)**	**IF group (*n* = 20)**	**BV group (*n* = 17)**	***p-*value**
Maternal age (years) [Median (range)]	33.0 (23.0–44.0)	34.0 (25.0–41.0)	32.5 (27.0–42.0)	0.271
Parity [Median (range)]	1.0 (0.0–4.0)	1.0 (0.0–3.0)	1.0 (0.0–3.0)	0.548
Abortion history [No (%)]	39 (22.5)	4 (20)	2 (11.1)	0.726
History of previous preterm birth [No (%)]	25 (14.5)	7 (20)	1 (5.6)	0.027*
Vaginal pH [Median (range)], (test No/total No)^a^	4.5 (4.0–6.0), 90/175	5 (4.0–5.0), 9/20	5 (4.0–6.5), 11/17	0.047^#^
Delivery weeks [Median (range)]	38.7 (18.0–41.4)	39.1 (33.1–40.6)	39.8 (21.6–41.0)	0.072
Preterm birth <37 weeks [No (%)]	22 (13)	4 (20)	2 (11.1)	0.656

The demographic characteristics were compared among the three groups using the non-parametric Kruskal–Wallis test followed by Mann–Whitney U-test (^#^p < 0.05 was statistically significant) and the Chi-square test (*p < 0.05 was statistically significant). No, number. Vaginal pH was obtained in 110 subjects.

a *The total number of subjects for which pH was obtained/total number of subjects in each group*.

### Clinical Symptoms

The frequency of increased thin homogenous vaginal discharge and foul odor was significantly different among the three groups (Supplementary Table 1). However, the data was obtained for only 168 women as the remaining participants refused to reveal this information. Moreover, clue cells were only observed, via Gram staining, in three cases all of which were categorized into the BV group (Supplementary Table 1).

### Overall Sequencing Output

A total of 13,978,305 reads (65,935.40 reads per sample ± 24,349.23 reads) from the MiSeq platform were trimmed to remove chimeric sequences and ambiguous base calls using the Deblur workflow resulting in 10,970,991 reads (51,749.96 reads per sample ± 18,860.65 reads) being retained (78.49%) for microbiome analysis including alpha, and beta diversity, as well as classification. The average length of all reads was matched to the same size as 400 nt. Diversity analysis was conducted with the minimum sequence reads of 21,928 to avoid variables caused by sequence read differences between samples.

### Five Main Community State Types in the Vaginal Microbiota

**Figure 1:**
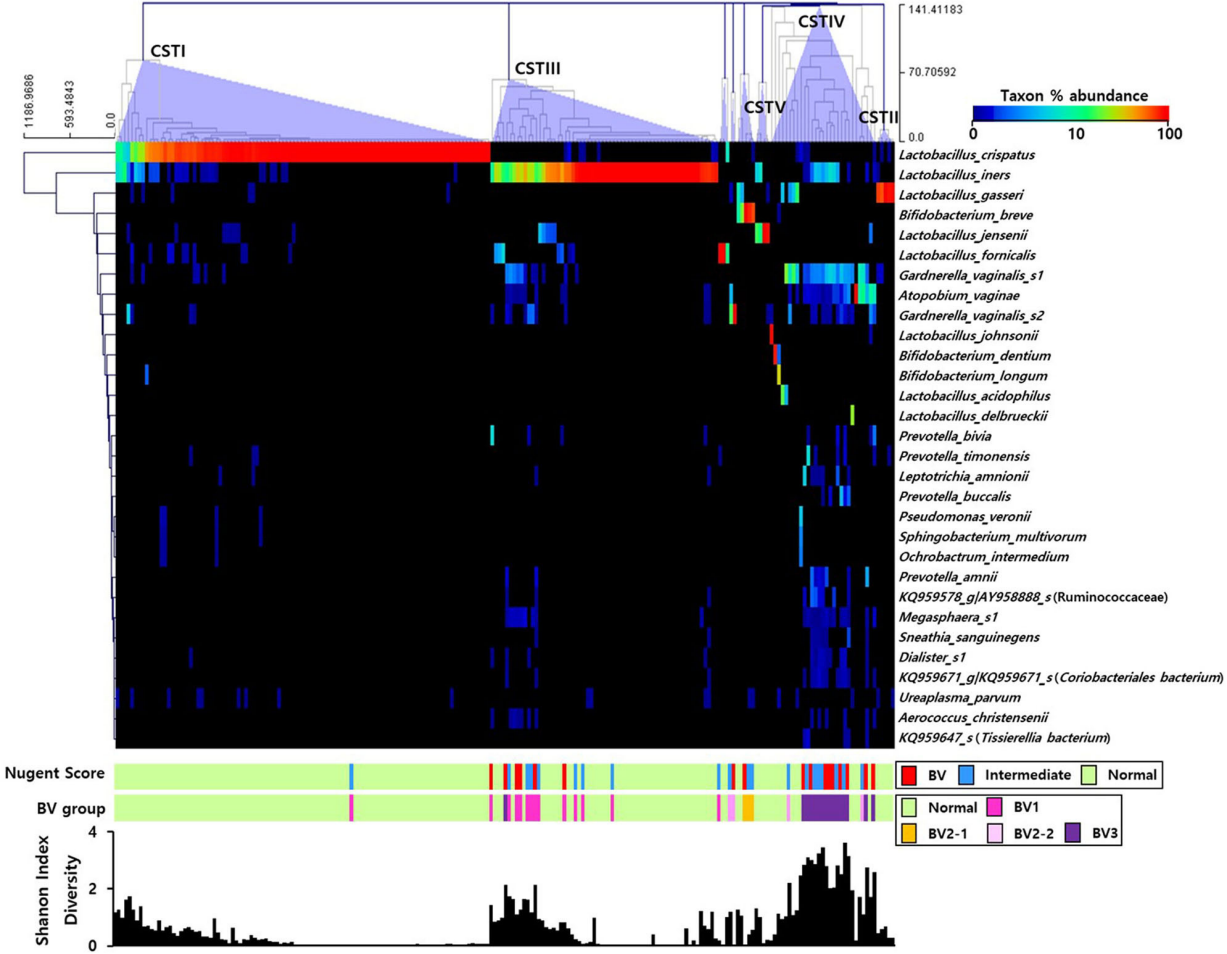
Heatmap of the relative abundances of vaginal microbial communities in the sampled pregnant Korean women. The dendrogram was constructed using the complete linkage clustering based on the Euclidian coefficient. The bars of the Nugent score and BV group indicate the vaginal condition of each sample. The graph at the bottom shows the Shannon diversity of each sample.

The RA of the top 30 OTUs in the pregnant Korean women were represented using a heatmap (Figure 1). Utilizing the clustering method with complete linkage based on Euclidian coefficients against both bacteria and subjects, we obtained five main CSTs, including CST I (*Lactobacillus crispatus*-dominant group, 48.1%; *n* = 102), CST II (*Lactobacillus gasseri-*dominant group, 2.4%; *n* = 5), CST III (*Lactobacillus iners* dominant group, 29.2%; *n* = 62), CST IV (diverse group, 13.7%; *n* = 29), and CST V (*Lactobacillus jensenii*-dominant group, 1.4%; *n* = 4). In addition, smaller groups (4.7%; *n* = 10), including *Lactobacillus formicalis, G. vaginalis*_s2, and *Bifidobacterium breve* were also obtained. *L. crispatus* and *L iners* accounted for the most substantial proportion of bacteria in CST I and CST III, respectively, and the Shannon index was shown to increase when their proportion was decreased. Additionally, the Nugent scoring system included the CST I (*L. crispatus*) cluster in the NF group, save for one sample, meanwhile the CST III (*L. iners*) cluster was only present in BV or IF cases at a low abundance.

### Relative Abundances of Different Genera and Species in the Vaginal Microbiota

**Figure 2:**
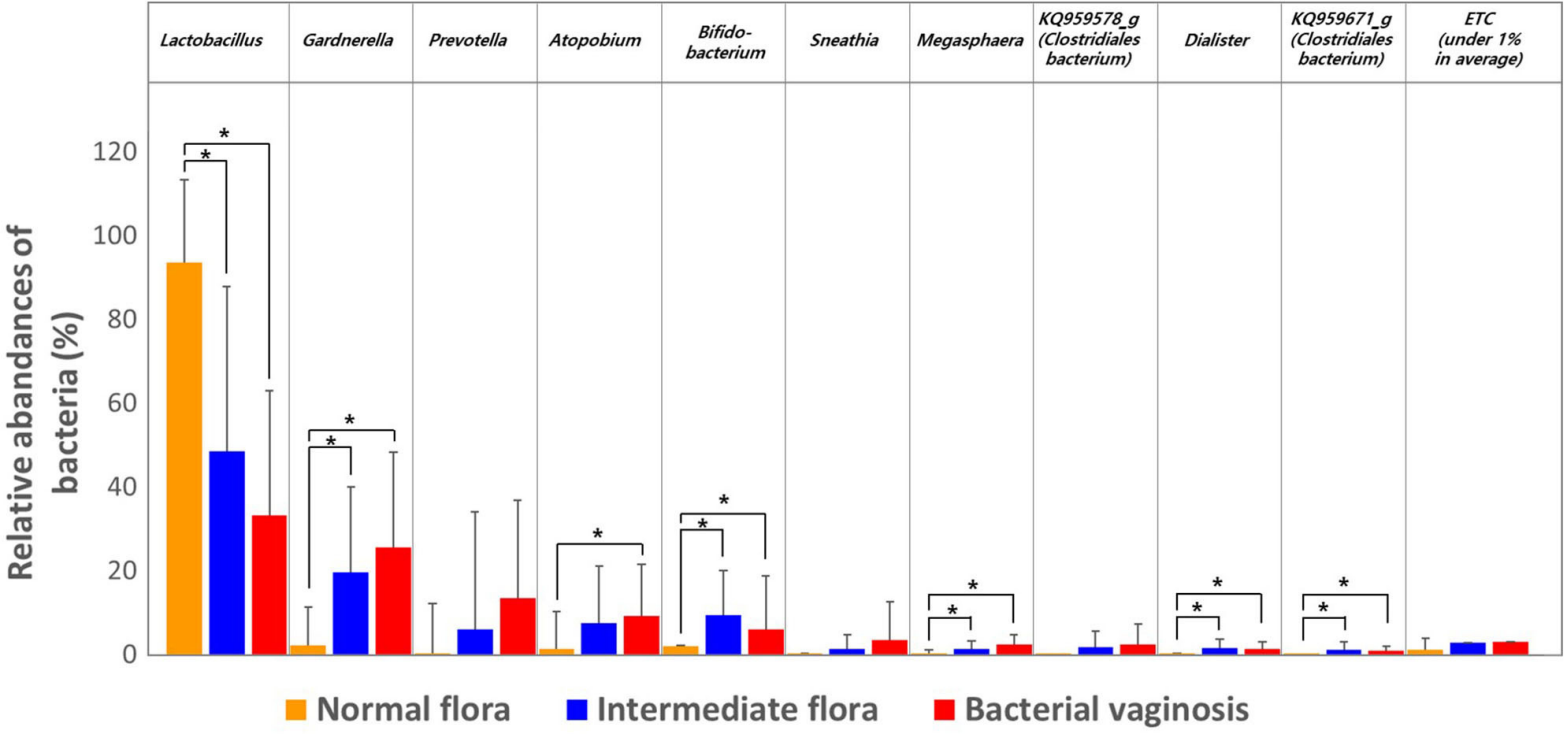
Relative abundances of the three groups, including normal flora, intermediate flora, and bacterial vaginosis. Average taxonomic compositions at the genus level among the subjects categorized into the three groups based on the Nugent score. The proportion of the bacterial composition was only presented at a cut-off of ≥1.0%. Error bars indicate standard deviation. Asterisk indicates significance between group (*p < 0.05) as determined using the Kruskal–Wallis test.

**Figure 3:**
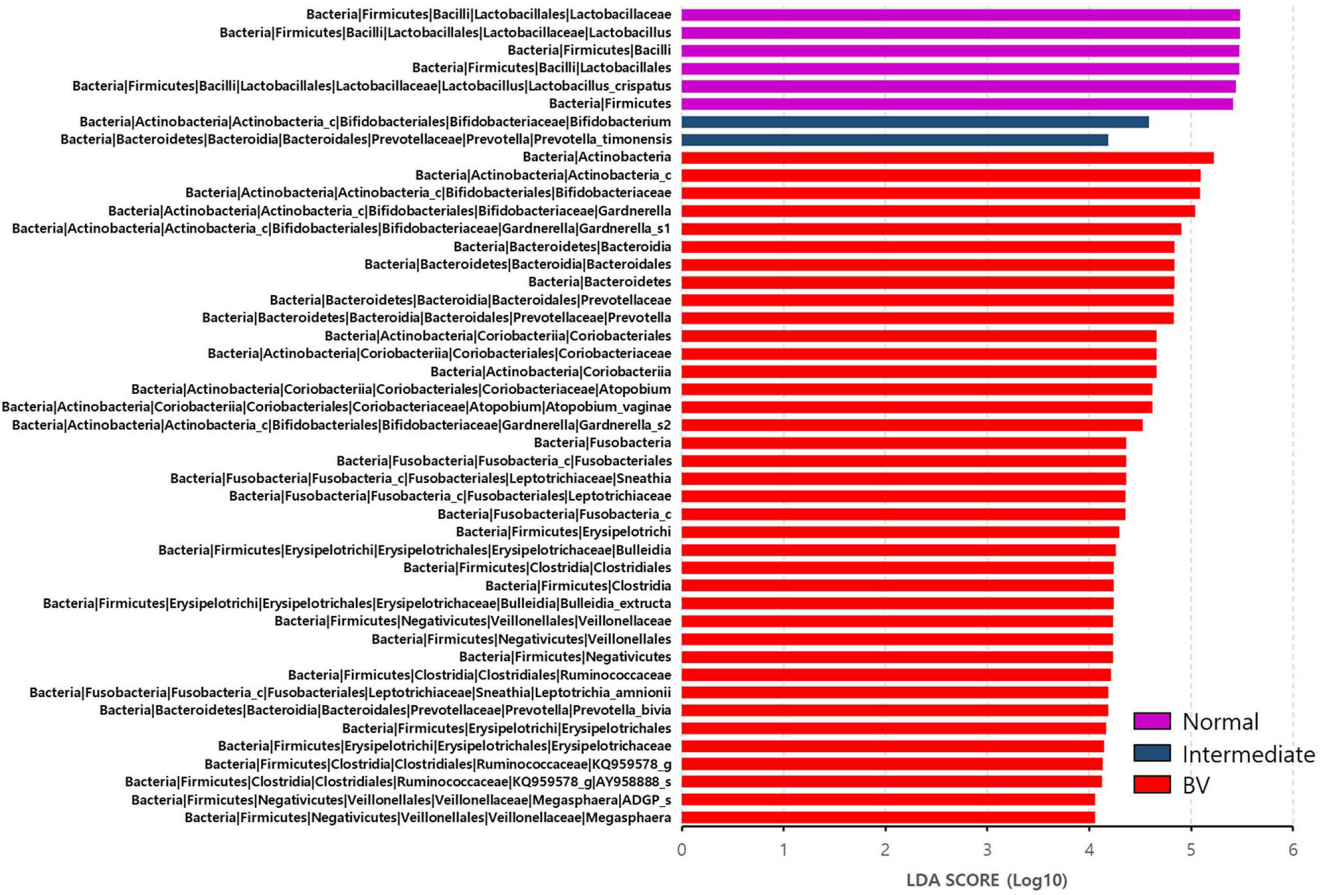
Differential abundance of the bacterial taxa in the three groups, including normal flora, intermediate flora, and bacterial vaginosis groups. Linear discriminant analysis effect size (LEfSe) analysis of the microbial profiles showed significant differences in the abundance of bacterial taxa of the three groups based on their Nugent scores. Normal flora, Intermediate flora, and BV are indicated by violet-, blue-, and red-colored bars, respectively. The minimum of the LDA score was set at four.

We then estimated the RA of different genera that accounted for over 1.0% of the total microbiota composition in the vaginal samples among the three groups obtained through Nugent scoring. Results show that the genus *Lactobacillus* was the most abundant in the NF group; while the IF and BV groups showed significantly higher abundances of the genera *Gardnerella, Prevotella, Atopobium*, as well as several others, compared with the NF group (*p* < 0.05; Figure 2). We also compared the RA of the three groups at the species level using the Kruskal–Wallis *H*-test with Bonferroni's correction. Thirty-four OTUs were shown to be significantly different among the three groups, including NF, IF, and BV (*p* < 0.05) (Supplementary Table 1). The NF group had higher RA of *L. crispatus* (54.87) and *L. reuteri* (0.08) among the three groups than their RA in IF (5.18, 0.02), and BV (0.02, 0.00) groups. Whereas a higher RA of *A. vaginae* was observed in IF and BV groups, (7.40 and 9.06, respectively) than the NF group (1.19). In addition, two *G. vaginalis* types had higher RA in the IF and BV groups than that in the NF group. RA of *G. vaginalis*_s1 was 12.27 and 16.74 in IF, and BV group, respectively, and that of *G. vaginalis*_s1 was 7.34, and 8.71. Moreover, the RAs of *Gemella asaccharolytica*, and another species (HM123928) of the *Bacteroidales* order, were higher in the IF and BV groups than the NF group. However, RAs of these two species were very low (Figure 3 and Supplementary Table 2).

**Figure 4:**
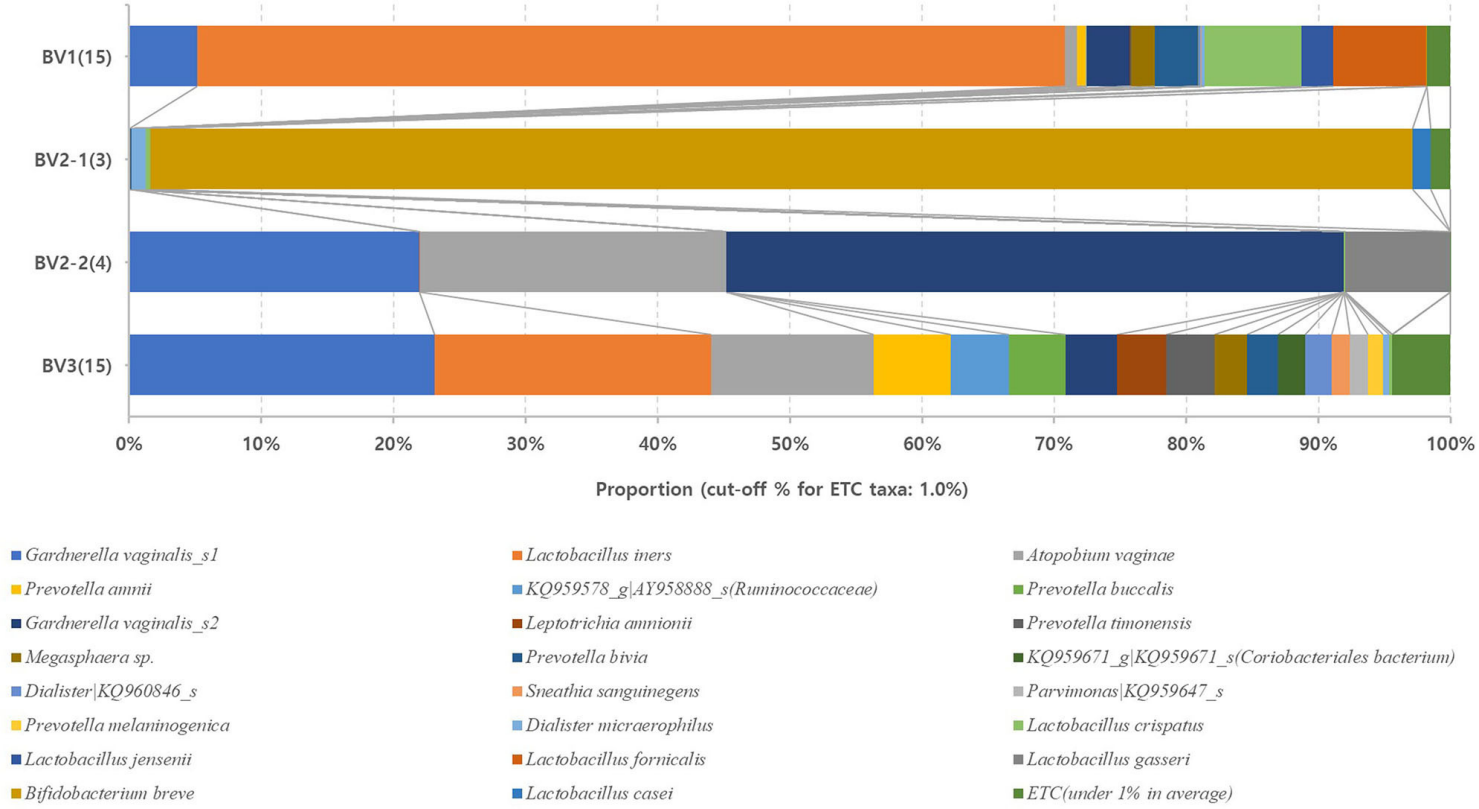
Relative abundances of the four reclassified BV groups. Average taxonomic compositions at the species level among the four BV groups. BV group (17 cases) and IF group (20 cases) were reclassified into four groups: BV1 (15 cases), BV2-1 (3 cases), BV2-2 (4 cases), and BV3 (15 cases). The proportion of the bacterial composition was only presented at a cut-off of ≥1.0%. #19 operative taxonomic units (OTUs); significantly different among the four groups (*p* < 0.05; Supplementary Table 2).

**Figure 5:**
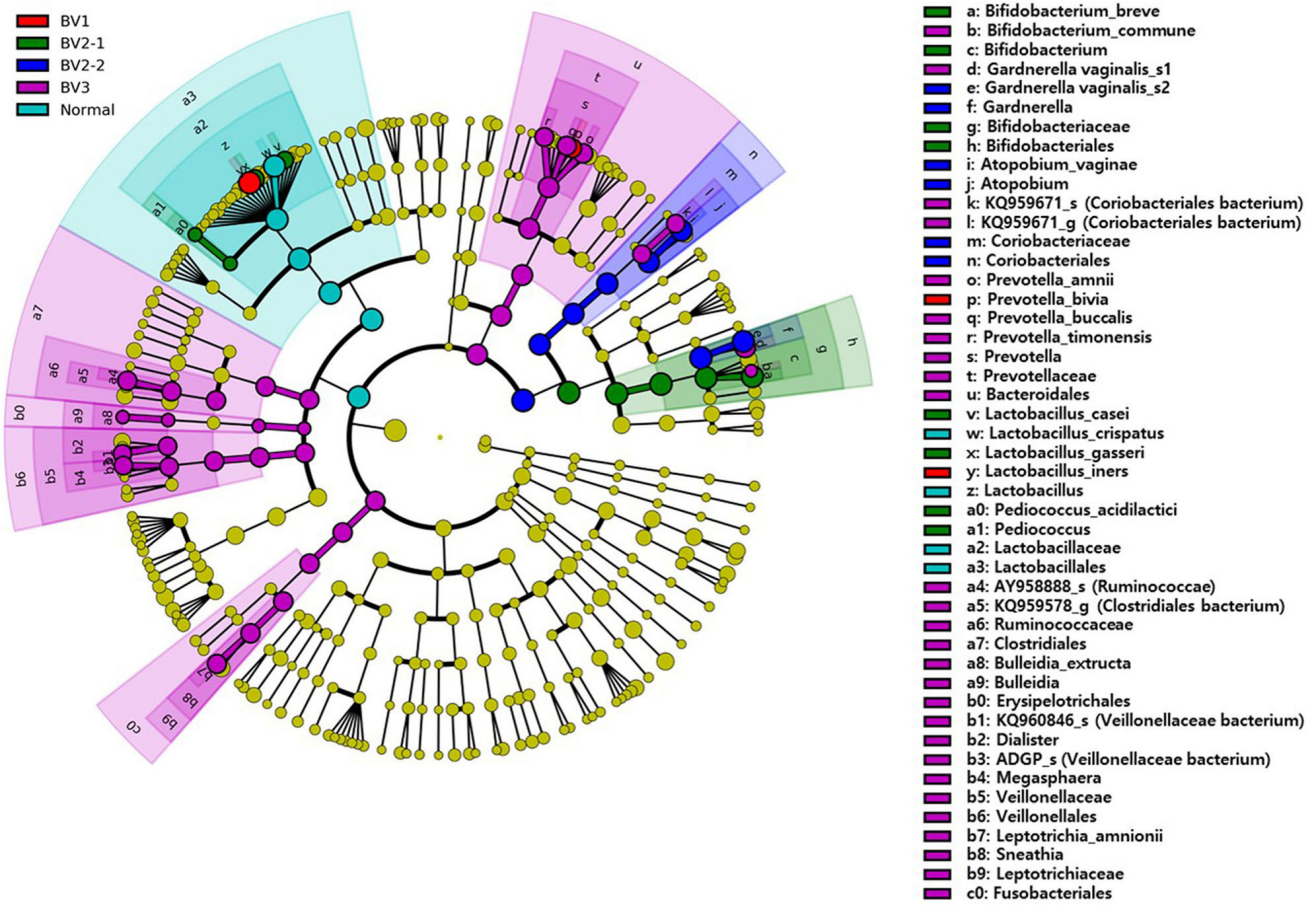
The cladogram based on linear discriminant analysis effect size (LEfSe) of the reclassified four BV groups and normal flora. Colors on the cladogram indicate; red (BV1), green (BV2-1), blue (BV2-2), violet (BV3), and sky blue (normal). Significantly abundant bacterial groups identified in this study are shown in the right-side list.

We reclassified the 37 subjects diagnosed as AVM (BV and IF microbiota) by the Nugent score using UPGMA clustering, into three groups. These groups were BV1 (*L. iners*-predominant) consisting of 15 subjects (40.5%; 15/37), BV2 (decreased alpha diversity and less *L. iners)* with 7 subjects (18.9%; 7/37), and BV3 (high alpha diversity; a mixed population of *G. vaginalis, L. iners*, and other bacteria) containing 15 subjects (40.5%; 15/37). Subsequently, the investigation of the vaginal microbiota of these three groups revealed that the BV1 group was dominated by *L. iners* (65.6%). The BV2 group showed two different microbiota compositional traits: the BV2-1 group comprised *B. breve* (95.5%) that produces lactic acid, whereas the BV2-2 group comprised *G. vaginalis* (68.6%) and *A. vaginae* (23.1%). The BV3 group comprised *G. vaginalis* (27.3%)*, L. iners* (20.9%), and other variable bacteria (Figure 4). We also compared the RA among the AVM groups at the species level using the Kruskal–Wallis *H*-test with Bonferroni's correction. Nineteen OTUs were shown to be significantly different among the AVM groups (*p* < 0.05). BV1 had the greatest abundance of *L. iners*, BV2-1 had the greatest abundance of *B. breve*, and BV2-2 had the greatest abundance of *G. vaginalis_*s2, or *G. vaginalis*_s1 and *A. vaginae*. Meanwhile, BV3 was composed of one species (KQ959671) from the family *Coriobacteriaceae*, one species (KQ960846) from the genus *Dialister*, and *Prevotella amnii* (Supplementary Table 3). A cladogram of the AVM and NF groups constructed using LEfSe showed that each group was located in different phylogenetic nodes. The NF group included most of the *Lactobacillus* spp., except for *L. iners*. BV2-1 included the *Bifidobacteriales* order with *B. breve*. BV2-2 included the *Coriobacteriales* order with *A. vaginae*, and BV3 included a variety of bacterial orders, including *Bacteroidales, Clostridiales, Erysipelotrichales, Veillonellales*, and *Fusobacteriales* (Figure 5).

Additionally, the Chi-square test for clinical symptoms, such as increased thin homogenous vaginal discharge (*p* = 0.331) and foul odor (*p* = 0.191) obtained through the questionnaire, did not show significant differences between the four AVM groups (Supplementary Table 1).

### Diversity Comparison: Species Richness and Diversity Indexes

**Figure 6:**
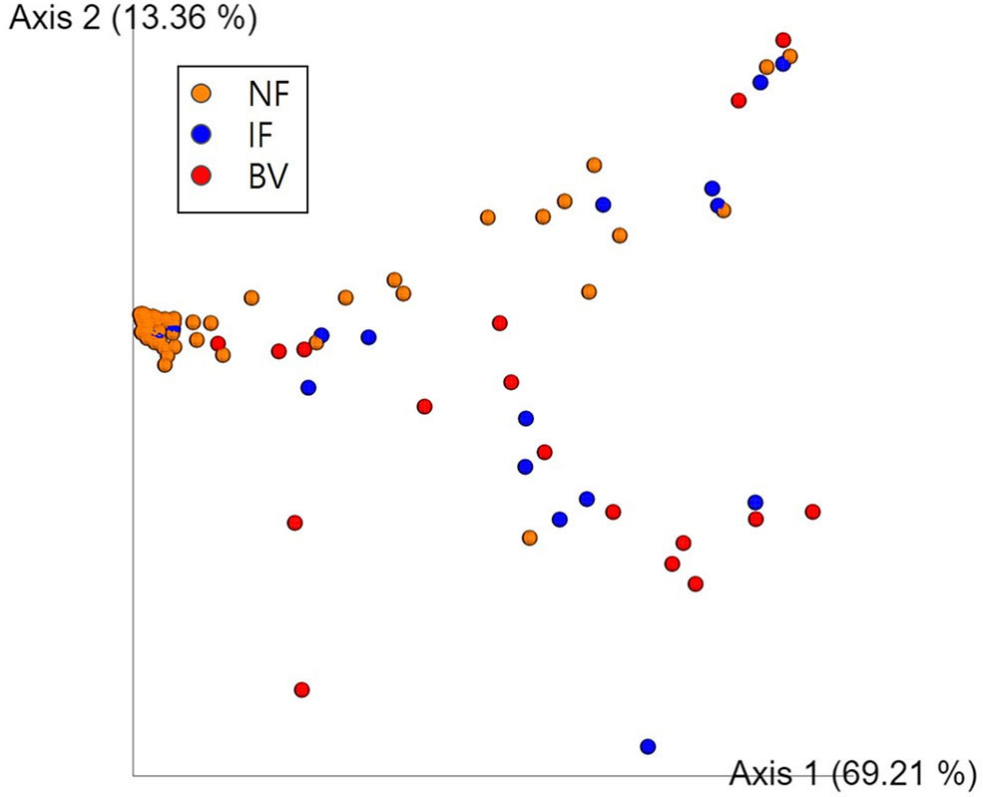
Principal coordinate analysis (PCoA) of the three groups based on weighted UniFrac distances. Each group was classified based on the Nugent score. NF, IF, and BV represents normal flora (orange), intermediate flora (blue), and bacterial vaginosis (red), respectively. Each axis 1 and 2 represents a Principal Component (PC), and the sum of the two-axes explanatory power is 82.57%.

Alpha diversity values for the observed OTUs, Faith's PD, and Shannon index revealed that the NF group differed significantly from both the IF and BV groups (*p* < 0.05). However, these valued did not differ significantly between the IF and BV groups (Shannon index, *p* = 0.411; Faith PD, *p* = 0.273; and observed OTUs, *p* = 0.211; Supplementary Table 4). The principal coordinate analysis (PCoA) indicated that the NF group was significantly different from the IF and BV groups (*p* < 0.05); however, there was no significant difference between the IF and BV groups (*p* = 0.25; Figure 6).

## Discussion

Only few studies have focused on pregnant Korean women to determine the vaginal microbial CSTs, nor the vaginal microbiota types associated with each Nugent score group (normal, intermediate, and BV microbiota). Moreover, since BV can have a devastating impact on pregnancy (6), it is critical to obtain a better understanding regarding the different vaginal microbiota associated with BV in women from various nationalities. In the present study, we analyzed the vaginal microbial composition of pregnant Korean women at the molecular level using NGS analysis. The NGS deep sequencing analysis followed by taxonomic classification was undertaken to explore the pathophysiology of AVM in 212 pregnant Korean women. These women were divided into three groups based on their Nugent scores, after which unique CSTs were identified for each group. Subsequently, characterization of the vaginal microbial composition at the genus and species level identified many genera and species prevalent in the vaginal samples. Phylogenetic analysis of the AVM group, consisting of individuals with IF and BV, divided the prevalent vaginal microbial composition into four groups, namely BV1, BV2-1, BV2-2, and BV3. Delineating the predominant composition of these four groups may prove useful to advance the current understanding regarding AVM pathophysiology in pregnant Korean women, specifically. Specifically, different vaginal microbiota compositions may have unique clinical significances. For instance, a high percentage of women with BV showed disease recurrence after antibiotic treatment, which may be associated with the specific vaginal microbiota composition common to BV (7–12).

Within these four AVM group we were able to delineate the taxonomic microbial characteristics of the vaginal microbial composition. Specifically, we determined that of the four AVM groups, BV3 had a high diversity index with 27.3% *G. vaginalis*, 20.9% *L. iners*, as well as other variable bacteria. The reduced abundance of *Lactobacillus* spp. along with the presence of diverse pathogenic microbiota in this group might lead to various clinical manifestations. However, the use of antibiotics or probiotics can restore the levels of *Lactobacillus*, thus improving the clinical symptoms (38–42). Alternatively, BV2-2 was found to be predominantly composed of *G. vaginalis_*s2 or *G. vaginalis_*s1, and *A. vaginae*. To date, these strains, especially *G. vaginalis*, are believed to function as the major microorganisms associated with BV, causing poor pregnancy outcomes, such as miscarriage and premature birth in pregnant women (9, 15–18). Here, we identified two types of *G. vaginalis* (*G. vaginalis_*s1 and *G. vaginalis_*s2) based on 16S rRNA gene sequencing. Meanwhile, different strains of *G. vaginalis* can be found in the “healthy” and “BV” state with a total of 13 different *Gardnerella* species proposed in a recent study (43), however, analysis at the specific gene or whole-genome level is required to estimate the full diversity of *Gardnerella* strains. Therefore, further studies are needed to delineate the relationships between the different strains of *G. vaginalis* and their implicated clinical symptoms. Moreover, within the BV2-1 group, *B. breve* was determined to be a predominant species, which was reported to be the dominant species in the guts of breast-fed infants (44). This species is also known as a probiotic responsible for protective effects in the intestine (44), however, there are few reports on its activity in the vagina. Since the clinical results of this vaginal microbial group are not yet known, further studies are needed to investigate the relationship between the BV2-1 group and clinical symptoms and to understand its clinical significance. Lastly, BV1 is the *L. iners*-predominant group. *L. iners* is a unique organism that is involved in both “healthy” and BV states (45). Unlike other *Lactobacillus* spp., *L. iners* can produce a cytolysin called inerolysin (similar to vaginolysin produced by *G. vaginalis*), indicating its virulence during BV development (46). Therefore, further studies for *L. iners* are needed to understand its clinical significance.

Additionally, molecular analysis of the V3–V4 region of 16S rRNA identified a total of five CSTs, three of which were newly defined and occupied by few subjects; meanwhile CST I and CST III predominated. The findings of the present study were supported by several other studies, which have reported that CST I and CST III, as compared to other CSTs, are more abundant in pregnant women than non-pregnant women (1–3). These differences could be attributed to the hormonal changes, lack of menstruation, and changes in sexual behavior that occur during pregnancy (2, 3, 6), in addition to the effect of residential areas including foods (14–19).

Although the study performed by Ravel et al. (1) used a 454 pyrosequencing platform targeting the V1–V2 regions of the 16S rRNA gene to investigate vaginal CSTs, rather than a MiSeq system targeting the V3–V4 region, as was employed in our study, no significant differences were observed in the diversity indices at the phylum or genus levels between these two platforms in a microbiome study (47). Therefore, the CSTs identified in this study can be readily compared with those from previous studies that were obtained using this alternate methodology.

Molecular analysis of the current study also identified fewer species in the NF group compared to the IF and BV groups. The distance between the clusters with regards to beta diversity clearly distinguished the NF group from the other groups. In contrast, the IF and BV groups could not be distinguished from each other. Furthermore, the species richness and diversity index (alpha diversity) analysis identified 34 OTUs that differed significantly between the IF and NF groups, whereas none showed differences between the IF and BV groups. These results may be attributable to an increase in the frequency of CST I and CST III groups in the vaginal microbiota of pregnant women with NF, as reported by Romero et al. (2) and Walther-António et al. (3). In contrast, Hong et al. showed a higher alpha diversity in the BV group, compared to that in the NF group, whereas no difference was observed in alpha diversities between the NF and IF groups in non-pregnant Korean women (48). These differences may be associated with the some pregnant components such as pregnancy hormones (2, 3). However, further studies for them are needed because of no researches.

Furthermore, to investigate the vaginal microbial composition more precisely and understand the pathophysiology of AVM, we analyzed clinical symptoms, including an increase in thin homogenous vaginal discharge, and foul odor, among women in these four AVM groups. However, no statistically significant differences were observed, which may have been caused by the small number of cases in the different groups (BV1, 15; BV2-1, 3; BV2-2, 4; BV3, 15). Note, that we also did not perform the whiff test but rather obtained the information on these clinical symptoms exclusively from the questionnaire, which is a limitation to this study in terms of evaluating the relationship between the vaginal microbial composition of the AVM group and clinical symptoms. This may have also limited our capacity to effectively evaluate the relationship between the vaginal microbial composition and the associated clinical significance, such as preterm birth.

In summary, this study indicates that NGS, with both deep sequencing and taxonomic classification, can delineate the compositions of vaginal microbiota more precisely in pregnant Korean women than the Nugent scoring system. The microbial composition of the four AVM groups identified here could be useful to understand the pathophysiology; however, considering that the vaginal environment is a dynamic ecosystem that undergoes natural fluctuations in microbial composition (3), large-scale, longitudinal studies capable of comparing clinical symptoms between the AVM types, are warranted. Thus, our study provides a basis for further investigation of the pathophysiology of these vaginal disease conditions and could help to establish appropriate treatment guidelines.

## Data Availability Statement

The 16S rRNA gene data sets generated in this study were deposited in the EBI European Nucleotide Archive database (European Bioinformatics Institute, Cambridge, UK) under the accession number PRJEB34614.

## Ethics Statement

The studies involving human participants were reviewed and approved by the Institutional Review Board (IRB) of Eulji University Hospital (IRB no: 2017-07-007-002). The patients/participants provided their written informed consent to participate in this study.

## Author Contributions

KO and B-YK conceived and designed the study. CJ, ES, and SK helped in data acquisition. SL and HH analyzed and interpreted the data. SL, KO, and B-YK contributed to the writing of the manuscript. All authors have read and approved the final manuscript.

## Conflict of Interest

SL was employed by ILDONG Pharmaceutical Co., Ltd. B-YK and HH were employed by ChunLab, Inc. The remaining authors declare that the research was conducted in the absence of any commercial or financial relationships that could be construed as a potential conflict of interest.

## Abbreviations

AVM: abnormal vaginal microbiota
BV: bacterial vaginosis
CSTs: community state types
NF: normal flora
NGS: next-generation sequencing
IF: intermediate flora
OTUs: operative taxonomic units
PCoA: Principal coordinate analysis.
