# Supplementary material for: Community State Types of Vaginal Microbiota and Four Types of Abnormal Vaginal Microbiota in Pregnant Korean Women
Source: Front Public Health. 2020 Oct 22;8:507024. doi: 10.3389/fpubh.2020.507024 (PMC7642985; doi:10.3389/fpubh.2020.507024)
Supplement: Supplementary file 1 [file Table_1.DOCX]

Supplementary Material

**Supplementary Table S1.** Clinical symptoms recorded via questionnaire from the three groups using the Nugent scoring system

| Symptoms | NF group  (n = 141)  Median (range) | IF group  (n = 14)  Median (range) | BV group  (n = 13)  Median (range) | *p-*value |
| --- | --- | --- | --- | --- |
| Thin homogenous vaginal discharge  (case No (%)) | 10 (7.1%) | 0 (0%) | 4 (31.8%) | 0.007* |
| Foul odor  (case No (%)) | 20 (14.2%) | 1/14 (7.1%) | 4/13 (30.8%) | 0.195 |

The total number of subjects for whom the data was available through the questionnaire was 168. The symptoms of the subjects were analyzed by the Chi-square test (**p-*value < 0.05 was statistically significant).

1. **Supplementary Table S2.** Quantitative comparison of the taxonomic composition of vaginal microbiota among the three groups diagnosed using Nugent score

| Taxon name | Relative abundances (%) | | | *p*-value | Bonferroni correction (*p*-value) | | |
| --- | --- | --- | --- | --- | --- | --- | --- |
|  | NF | IF | BV |  | NF:IF | NF:BV | IF:BV |
| Bacteria\|Firmicutes\|Negativicutes\|Veillonellales\|Veillonellaceae\|Dialister\|Dialister_micraerophilus | 0.03 | 0.49 | 0.32 | 0.000 | **0.000** | **0.000** | 0.153 |
| Bacteria\|Firmicutes\|Negativicutes\|Veillonellales\|Veillonellaceae\|Dialister\|KQ960846_s | 0.01 | 0.91 | 0.93 | 0.000 | **0.000** | **0.000** | 0.101 |
| Bacteria\|Firmicutes\|Negativicutes\|Veillonellales\|Veillonellaceae\|Megasphaera\|ADGP_s | 0.12 | 1.18 | 2.37 | 0.000 | **0.030** | **0.000** | **0.000** |
| Bacteria\|Firmicutes\|Bacilli\|Lactobacillales\|Aerococcaceae\|Aerococcus\|Aerococcus_christensenii | 0.02 | 0.39 | 0.67 | 0.000 | **0.001** | **0.000** | **0.004** |
| Bacteria\|Actinobacteria\|Coriobacteriia\|Coriobacteriales\|Coriobacteriaceae\|KQ959671_g\|KQ959671_s | 0.00 | 1.04 | 0.77 | 0.000 | **0.000** | **0.000** | **0.023** |
| Bacteria\|Actinobacteria\|Coriobacteriia\|Coriobacteriales\|Coriobacteriaceae\|Atopobium\|Atopobium_vaginae | 1.19 | 7.40 | 9.06 | 0.000 | **0.000** | **0.000** | 0.064 |
| Bacteria\|Firmicutes\|Tissierellia\|Tissierellales\|Peptoniphilaceae\|Parvimonas\|KQ959647_s | 0.00 | 0.58 | 0.60 | 0.000 | **0.001** | **0.000** | 0.052 |
| Bacteria\|Firmicutes\|Clostridia\|Clostridiales\|Ruminococcaceae\|KQ959578_g\|AY958888_s | 0.00 | 1.59 | 2.33 | 0.000 | **0.025** | **0.000** | **0.010** |
| Bacteria\|Fusobacteria\|Fusobacteria_c\|Fusobacteriales\|Leptotrichiaceae\|Sneathia\|Sneathia_sanguinegens | 0.01 | 0.25 | 1.07 | 0.000 | 0.080 | **0.000** | 0.115 |
| Bacteria\|Actinobacteria\|Actinobacteria_c\|Bifidobacteriales\|Bifidobacteriaceae\|Gardnerella\|Gardnerella_vaginalis_s2 | 0.47 | 7.34 | 8.71 | 0.000 | **0.000** | **0.000** | 1.000 |
| Bacteria\|Actinobacteria\|Actinobacteria_c\|Bifidobacteriales\|Bifidobacteriaceae\|Gardnerella\|Gardnerella_vaginalis_s1 | 1.67 | 12.27 | 16.74 | 0.000 | **0.000** | **0.000** | 1.000 |
| Bacteria\|Actinobacteria\|Actinobacteria_c\|Bifidobacteriales\|Bifidobacteriaceae\|Bifidobacterium\|Bifidobacterium_commune | 0.00 | 0.00 | 0.01 | 0.000 | 0.140 | **0.000** | 0.445 |
| Bacteria\|Firmicutes\|Bacilli\|Lactobacillales\|Lactobacillaceae\|Lactobacillus\|Lactobacillus_crispatus | 54.87 | 5.18 | 0.20 | 0.000 | **0.000** | **0.000** | 1.000 |
| Bacteria\|Fusobacteria\|Fusobacteria_c\|Fusobacteriales\|Leptotrichiaceae\|Sneathia\|Leptotrichia_amnionii | 0.02 | 0.96 | 2.40 | 0.000 | 0.080 | **0.000** | 0.115 |
| Bacteria\|Tenericutes\|Mollicutes\|Mycoplasmatales\|Mycoplasmataceae\|Mycoplasma_g4\|Mycoplasma_hominis | 0.04 | 0.23 | 0.01 | 0.000 | **0.046** | **0.000** | 0.549 |
| Bacteria\|Bacteroidetes\|Bacteroidia\|Bacteroidales\|Prevotellaceae\|Prevotella\|Prevotella_amnii | 0.00 | 1.01 | 4.87 | 0.000 | 0.510 | **0.000** | 0.074 |
| Bacteria\|Firmicutes\|Erysipelotrichi\|Erysipelotrichales\|Erysipelotrichaceae\|Bulleidia\|Bulleidia_extructa | 0.00 | 0.00 | 0.02 | 0.000 | 0.229 | **0.000** | 0.236 |
| Bacteria\|Bacteroidetes\|Bacteroidia\|Bacteroidales\|Prevotellaceae\|Prevotella\|Prevotella_bivia | 0.03 | 1.02 | 3.64 | 0.001 | **0.001** | 0.217 | 0.728 |
| Bacteria\|Bacteroidetes\|Bacteroidia\|Bacteroidales\|Prevotellaceae\|Prevotella\|JRNC_s | 0.01 | 0.62 | 0.06 | 0.001 | **0.010** | **0.044** | 1.000 |
| Bacteria\|Bacteroidetes\|Bacteroidia\|Bacteroidales\|Prevotellaceae\|Prevotella\|Prevotella_melaninogenica | 0.00 | 0.00 | 1.07 | 0.002 | 1.000 | **0.001** | 0.082 |
| Bacteria\|Bacteroidetes\|Bacteroidia\|Bacteroidales\|Prevotellaceae\|Prevotella\|Prevotella_timonensis | 0.03 | 2.79 | 0.24 | 0.002 | **0.005** | 0.207 | 1.000 |
| Bacteria\|Firmicutes\|Bacilli\|Lactobacillales\|Lactobacillaceae\|Lactobacillus\|Lactobacillus_reuteri | 0.08 | 0.020.02 | 0.00 | 0.003 | **0.026** | **0.042** | 1.000 |
| Bacteria\|Firmicutes\|Bacilli\|Bacillales\|Gemella_f\|Gemella\|Gemella_asaccharolytica | 0.00 | 0.01 | 0.00 | 0.003 | **0.002** | 1.000 | **0.032** |
| Bacteria\|Bacteroidetes\|Bacteroidia\|Bacteroidales\|EU845084_f\|GU302773_g\|HM123928_s | 0.00 | 0.00 | 0.13 | 0.003 | 1.000 | **0.002** | **0.028** |
| Bacteria\|Actinobacteria\|Actinobacteria_c\|Actinomycetales\|Actinomycetaceae\|Mobiluncus\|Mobiluncus_mulieris | 0.00 | 0.23 | 0.00 | 0.008 | **0.006** | 1.000 | 0.082 |
| Bacteria\|Actinobacteria\|Coriobacteriia\|Coriobacteriales\|Coriobacteriaceae\|Atopobium\|AEDQ_s | 0.00 | 0.06 | 0.00 | 0.008 | **0.006** | 1.000 | 0.082 |
| Bacteria\|Firmicutes\|Bacilli\|Lactobacillales\|Lactobacillaceae\|Lactobacillus\|Lactobacillus_casei | 0.00 | 0.21 | 0.00 | 0.008 | **0.006** | 1.000 | 0.082 |
| Bacteria\|Actinobacteria\|Actinobacteria_c\|Actinomycetales\|Actinomycetaceae\|Mobiluncus\|Mobiluncus_curtisii | 0.00 | 0.00 | 0.01 | 0.010 | 0.450 | **0.015** | 0.779 |
| Bacteria\|Firmicutes\|Clostridia\|Clostridiales\|Peptostreptococcaceae\|Peptostreptococcus\|Peptostreptococcus_anaerobius | 0.00 | 0.06 | 0.17 | 0.010 | **0.013** | 0.568 | 0.913 |
| Bacteria\|Bacteroidetes\|Bacteroidia\|Bacteroidales\|Porphyromonadaceae\|Porphyromonas\|Porphyromonas_asaccharolytica | 0.00 | 0.08 | 0.02 | 0.016 | 0.809 | **0.018** | 0.551 |
| Bacteria\|Firmicutes\|Tissierellia\|Tissierellales\|Peptoniphilaceae\|Anaerococcus\|Anaerococcus_tetradius | 0.00 | 0.04 | 0.05 | 0.016 | 0.349 | **0.032** | 1.000 |
| Bacteria\|Firmicutes\|Negativicutes\|Veillonellales\|Veillonellaceae\|Megasphaera\|AFUG_s | 0.00 | 0.03 | 0.00 | 0.017 | 1.000 | **0.016** | 0.075 |
| Bacteria\|Firmicutes\|Clostridia\|Clostridiales\|Ruminococcaceae\|Mageeibacillus\|Mageeibacillus_indolicus | 0.00 | 0.01 | 0.44 | 0.027 | **0.039** | 0.624 | 1.000 |
| Bacteria\|Bacteroidetes\|Bacteroidia\|Bacteroidales\|Prevotellaceae\|Prevotella\|Prevotella_buccalis | 0.00 | 0.52 | 3.42 | 0.040 | 1.000 | **0.036** | 0.383 |

All relative abundance data for species that were significantly different are presented in this table, regardless of their value (e.g. values below 1.0%). Relative abundance was analyzed using the Kruskal-Wallis H test followed by the Mann-Whitney U test using Bonferroni correction to adjust the probability. Bonferroni-adjusted *p* values were used (*p* < 0.05).

**Supplementary Table S3.** Quantitative comparison of taxonomic composition of vaginal microbiota among the four groups with abnormal flora using clustering program

| **Taxon name** | **Relative abundances (%)** | | | | | | | ***p*-value** | | **Bonferroni correction (*p*-value)** | | | | | |
| --- | --- | --- | --- | --- | --- | --- | --- | --- | --- | --- | --- | --- | --- | --- | --- |
|  | **BV1** | **BV2-1** | | **BV2-2** | | **BV3** | |  |  | **BV1:**  **BV2-1** | **BV1:**  **BV2-2** | **BV1:**  **BV3** | **BV2-1:**  **BV2-2** | **BV2-1:**  **BV3** | **BV2-2:**  **BV3** |
| Bacteria\|Actinobacteria\|Coriobacteriia\|Coriobacteriales\|Coriobacteriaceae\|KQ959671_g\|KQ959671_s | 0.04 | | 0.00 | | 0.00 | | 2.08 | | 0.000 | 1.000 | 1.000 | **0.000** | 1.000 | **0.003** | 0.107 |
| Bacteria\|Firmicutes\|Negativicutes\|Veillonellales\|Veillonellaceae\|Dialister\|KQ960846_s | 0.16 | | 0.00 | | 0.01 | | 1.99 | | 0.000 | 0.851 | 1.000 | **0.005** | 1.000 | **0.001** | 0.199 |
| Bacteria\|Firmicutes\|Bacilli\|Lactobacillales\|Lactobacillaceae\|Lactobacillus\|Lactobacillus_iners | 65.64 | | 0.06 | | 0.05 | | 20.93 | | 0.000 | **0.001** | 0.139 | **0.044** | 1.000 | 0.245 | 1.000 |
| Bacteria\|Actinobacteria\|Coriobacteriia\|Coriobacteriales\|Coriobacteriaceae\|Atopobium\|Atopobium_vaginae | 0.90 | | 0.02 | | 23.17 | | 12.29 | | 0.001 | 1.000 | 1.000 | **0.000** | 1.000 | 0.372 | 1.000 |
| Bacteria\|Firmicutes\|Tissierellia\|Tissierellales\|Peptoniphilaceae\|Fenollaria\|LN898229_s | 0.00 | | 0.00 | | 0.00 | | 0.00 | | 0.001 | 1.000 | **0.000** | 1.000 | **0.002** | 1.000 | **0.000** |
| Bacteria\|Firmicutes\|Tissierellia\|Tissierellales\|Peptoniphilaceae\|Parvimonas\|KQ959647_s | 0.01 | | 0.00 | | 0.00 | | 1.35 | | 0.001 | 1.000 | 1.000 | **0.005** | 1.000 | **0.030** | 0.330 |
| Bacteria\|Fusobacteria\|Fusobacteria_c\|Fusobacteriales\|Leptotrichiaceae\|Sneathia\|Leptotrichia_amnionii | 0.06 | | 0.00 | | 0.00 | | 3.70 | | 0.003 | 1.000 | 1.000 | **0.007** | 1.000 | 0.057 | 0.459 |
| Bacteria\|Proteobacteria\|Betaproteobacteria\|Burkholderiales\|Ralstonia_f\|Ralstonia\|Ralstonia_pickettii | 0.00 | | 0.02 | | 0.00 | | 0.00 | | 0.004 | **0.005** | 1.000 | 1.000 | 0.223 | **0.004** | 1.000 |
| Bacteria\|Firmicutes\|Clostridia\|Clostridiales\|Ruminococcaceae\|KQ959578_g\|AY958888_s | 0.04 | | 0.00 | | 0.00 | | 4.42 | | 0.005 | 1.000 | 1.000 | **0.017** | 1.000 | 0.056 | 0.453 |
| Bacteria\|Firmicutes\|Negativicutes\|Veillonellales\|Veillonellaceae\|Megasphaera\|ADGP_s | 1.73 | | 0.00 | | 0.00 | | 2.47 | | 0.006 | 0.545 | 1.000 | 0.361 | 1.000 | **0.013** | 0.219 |
| Bacteria\|Actinobacteria\|Actinobacteria_c\|Bifidobacteriales\|Bifidobacteriaceae\|Gardnerella\|Gardnerella_vaginalis_s1 | 5.18 | | 0.00 | | 21.96 | | 23.11 | | 0.007 | 1.000 | 1.000 | **0.009** | 1.000 | 0.117 | 1.000 |
| Bacteria\|Actinobacteria\|Actinobacteria_c\|Bifidobacteriales\|Bifidobacteriaceae\|Bifidobacterium\|Bifidobacterium_breve | 0.01 | | 95.50 | | 0.00 | | 0.00 | | 0.008 | 0.126 | 1.000 | 0.912 | 0.234 | **0.004** | 1.000 |
| Bacteria\|Fusobacteria\|Fusobacteria_c\|Fusobacteriales\|Leptotrichiaceae\|Sneathia\|Sneathia_sanguinegens | 0.06 | | 0.00 | | 0.00 | | 1.41 | | 0.008 | 1.000 | 1.000 | **0.019** | 1.000 | 0.097 | 0.601 |
| Bacteria\|Firmicutes\|Bacilli\|Lactobacillales\|Lactobacillaceae\|Lactobacillus\|Lactobacillus_gasseri | 0.02 | | 0.00 | | 7.93 | | 0.00 | | 0.009 | 0.153 | 1.000 | 0.865 | 0.254 | **0.006** | 1.000 |
| Bacteria\|Bacteroidetes\|Bacteroidia\|Bacteroidales\|Prevotellaceae\|Prevotella\|JRNC_s | 0.00 | | 0.00 | | 0.00 | | 0.84 | | 0.015 | 1.000 | 1.000 | **0.031** | 1.000 | 0.143 | 0.735 |
| Bacteria\|Bacteroidetes\|Bacteroidia\|Bacteroidales\|Prevotellaceae\|Prevotella\|Prevotella_amnii | 0.68 | | 0.00 | | 0.00 | | 5.84 | | 0.021 | 1.000 | 1.000 | **0.047** | 1.000 | 0.160 | 0.781 |
| Bacteria\|Proteobacteria\|Betaproteobacteria\|Neisseriales\|Neisseriaceae\|FM873692_g\|FM873692_s | 0.00 | | 0.00 | | 0.01 | | 0.00 | | 0.026 | 1.000 | 0.063 | 1.000 | **0.045** | 1.000 | **0.017** |
| Bacteria\|Firmicutes\|Bacilli\|Lactobacillales\|Aerococcaceae\|Aerococcus\|Aerococcus_christensenii | 0.44 | | 0.00 | | 0.00 | | 0.81 | | 0.031 | 0.460 | 1.000 | 1.000 | 1.000 | **0.050** | 0.431 |
| Bacteria\|Firmicutes\|Bacilli\|Lactobacillales\|Enterococcaceae\|Tetragenococcus\|Tetragenococcus_halophilus | 0.00 | | 0.00 | | 0.00 | | 0.00 | | 0.042 | 0.137 | 1.000 | 1.000 | 0.506 | **0.029** | 1.000 |

All relative abundance data for species that were significantly different are presented in this table, regardless of their value (e.g. values below 1.0 %). Relative abundance was analyzed using the Kruskal-Wallis H test followed by the Mann-Whitney *U* test using Bonferroni correction to adjust the probability. Bonferroni-adjusted *p* values were used (*p* < 0.05).

**Supplementary Table S4.** Statistical comparison of alpha-diversity index among NF, IF, and BV groups

| **α-diversity index** | **Group 1** | **Group 2** | **H** | ***p*-value** | ***q*-value** |
| --- | --- | --- | --- | --- | --- |
| **observed_OTUs*** | BV (n = 17) | Intermediate (n = 20) | 1.564 | 0.211 | 0.211 |
|  | BV (n = 17) | Normal (n = 175) | 22.726 | 0.000 | **0.000** |
|  | Intermediate (n = 20) | Normal (n = 175) | 13.133 | 0.000 | **0.000** |
| **Faith_pd** | BV (n = 17) | Intermediate (n = 20) | 1.204 | 0.273 | 0.273 |
|  | BV (n = 17) | Normal (n = 175) | 21.299 | 0.000 | **0.000** |
|  | Intermediate (n=20) | Normal (n = 175) | 11.477 | 0.001 | **0.001** |
| **Shannon** | BV (n = 17) | Intermediate (n = 20) | 0.677 | 0.411 | 0.411 |
|  | BV (n = 17) | Normal (n = 175) | 27.808 | 0.000 | **0.000** |
|  | Intermediate (n = 20) | Normal (n = 175) | 17.458 | 0.000 | **0.000** |

*OTUs, operative taxonomic units
